# Prediction of geopolymer pumpability and setting time for well zonal isolation - Using machine learning and statistical based models

**DOI:** 10.1016/j.heliyon.2023.e17925

**Published:** 2023-07-11

**Authors:** Anis Hoayek, Mahmoud Khalifeh, Hassan Hamie, Bassam El-Ghoul, Rania Zogheib

**Affiliations:** a Mines Saint-Etienne, Universite Clermont Auvergne, CNRS, UMR 6158 LIMOS, Institut Henri Fayol, F- 42023, Saint-Etienne, France; b University of Stavanger, Faculty of Science and Technology, Department of Energy and Petroleum Eng., 4036 Stavanger, Norway; c Vienna University of Technology, Institute of Energy Systems and Electrical Drives, Energy Economics Group (EEG), Austria; d Saint Petersburg Mining University, Faculty of Oil and Gas Engineering, Saint Petersburg, Russia; e Holly Spirit University of Kaslik Lebanon, Faculty of Science and Technology, Dept. of Applied Mathematics and Engineering, Lebanon

**Keywords:** Geopolymers, Well cementing, Modeling pumpability, Logistic regression, Supportive vector machine, ANN

## Abstract

In academia, geopolymers/alkali activated materials are becoming increasingly popular, as researchers are exploring a substitute for Portland cement, that is cost-effective, merchantable, and potent. One such potential-seeking sector is the use of geopolymers in the oil and gas industry for well cementing zonal isolation operations. Though it is yet, to be implemented in the field, there has been a few researchers, although not so many in numbers, that conducted geopolymer lab-tests (controlled and expensive environment). Pushed by the fact that any product must be tested under a vast space of external conditions, before being commercialized; the authors wish to address the gap, by applying a variety of prediction models, with the aim to produce accurate results, while relying on a limited set of experimental data.

Binary/multi thresholds classification (logistic/probit, decision tree, random forest, SVM), as well as regression and continuous models (multi linear regression, neural networks, among others) are used to predict an important property of the geopolymers (pumpability). This is important, as despite the proven strong performance of such models in other areas, the novelty of the product/subject, uniqueness/insufficiency of the data and the high sensitivity in the behavior of the geopolymers (especially for the pumpability property) subject to slight changes to the chemical mixture design, accurate results and validation is yet to be tested, and most importantly, the ability of the models to generalize. The study uncovers that Decision Tree model provides a simple and intuitive way to understand the behavior of the geopolymers, subject to a variety of external conditions, and can in fact, be used by future users, to accurately predict the pumpability conditions. Although present, inaccurate/poor predictions (false positive) with high operational risk (defined as a geopolymer not reaching its polycondensation phase) have very low probability of occurrence.

## Highlights

• Predicting pumpability of granite-based geopolymers by use of mathematical models.
• Both continuous (regression) and discrete (classification) methods were used.
• Non-linear models extract complex pattern from limited data and generalize as well.
• Decision Tree provides an intuitive way to predict the behavior of the Geopolymers.

## 1 Introduction

Geopolymers are an emerging class of inorganic polymers with cementitious properties that offer a more environmentally friendly alternative to Ordinary Portland Cement (OPC), a highly demanded product in the market. While numerous scholars have focused on the chemistry of geopolymers and alkali activated materials [[1], [2], [3]], others have developed mathematical methods to predict and optimize the behavior of geopolymers. These prediction methods include regression, fuzzy logic, and Artificial Neural Network (ANN) [[4], [5], [6]]. While most of these publications investigate the prediction performance of geopolymer properties using a variety of mathematical models to be used in the field of construction and building materials, few scholars focused on geopolymer application in well cementing for Oil and Gas industry [7]. Despite this, computational modeling methods are widely used throughout the oil and gas value chain, with some products being commercialized, particularly in the field of reservoir engineering.

In academia, geopolymers/alkali activated materials are becoming increasingly popular, as researchers are exploring a potential cheaper and more efficient substitute to the Portland cement; one such potential-seeking sector is the use of geopolymers in oil and gas industry for well cementing zonal isolation operations. However, careful consideration of critical parameters such as the dissolution and oligomerization phases is required to ensure that the geopolymer slurry retains its rheological properties in harsh downhole well conditions, such as the geothermal gradient. Time is also a crucial factor, as the rheological properties must be preserved during the time it takes to pump the product from the ground to the desired depth. As a rule of thumb, this pumping time should be at least 4 h at the target temperature, not including the ramp-up rate and time required for the temperature to reach the target level. The complexity gets multiplied due to the unique nature of each well, which can result in varying pumping times. As much as it is labeled as a challenge, however with careful analysis, the use of geopolymers offer many economic and environmental benefits, and therefore an accurate prediction of the pumping time helps engineers, benefit of such a product. At the same time, the careful use of such a product, will reduce the risk of geopolymer setting inside casing or drillpipe to a great extent, while controlling for high temperatures and conserving the rheological properties of the slurry.

Laboratory work and experiments are considered to have a controlled set-up condition, therefore offer limited number of measurements. This is mainly due to high costs, complicated instrumentation setup, time-consuming experiments, among others. Unfortunately, geopolymers use in well cementing operations have not been implemented in the field yet, and the development is still conducted in controlled environmental sets, such as small/medium-scale laboratories, therefore unlike lab data abundance, field data is scarce. This being said, means that there is a need to understand and explore the behavior and change in proprieties of geopolymers while being exposed to extreme field conditions. A modeler requires to have more measurements, and therefore more data to analyze. In an effort to make the work automated and get fast analysis, without jeopardizing accuracy, the authors will apply a variety of prediction models, that take into account the various external conditions scenarios, and focus on the prediction of geopolymer pumping time.

The geopolymer mix designs to be used in this study, for simulation purposes, are granite-based geopolymers developed for downhole applications [[8], [9], [10]]. Chemistry, rheological properties and mechanical performance of these geopolymers have comprehensively been examined and published [[11], [12], [13], [14], [15]]. However, as mentioned previously, the experimental work is time-consuming, costly and engages access to hardware resources. This challenge may be surmounted with computing systems.

This work aims to review and evaluate performance of different modeling approaches, while using a limited set of granite-based geopolymers generated experimental data, for simulation, empirical and testing purposes. The experimental generated data is tested under well operations conditions according to API 10B-2 [16]. The objective is to identify advantages and shortcomings of the proposed models to predict the pumpability properties of the geopolymers and their polycondensation phase.

## 2 Knowledge and background on modeling

Accurate measures of concrete mix mechanical properties (such as, but not limited to, volume change, permeability, ductility, and long-term durability) is conducted in laboratories. There are numerous experimental results published in different platforms. A less accurate technique makes use of the laboratory results, and with the use of statistical methods and assumptions, develop models that can anticipate the behavior, to allow users and practitioners to evaluate quickly the results and subsequently assign probabilities of mechanical failure. These models are expanding and being developed further, algorithm performance is being enhanced, and with the use of advanced computing power and hardware capabilities, efficient machine learning technique are being deployed.

In parallel, cement is being replaced in some important industries, most notably in the oil industry, such as the case of geopolymers being developed for zonal isolation application, as it has proved to provide superior mechanical properties when compared to conventional Portland cement. Coupled with the ease of production, low cost, low carbon footprint and abundance, it can prove to be a good substitute in the oil industry.

With the growing interest in geopolymers, advancement in machine learning model performance, researchers shall make use of experimental data, in order to develop models with strong prediction ability. Despite the strong performance of such models, the novelty of the subject, uniqueness/insufficiency of the data and the high sensitivity in the behavior of the geopolymers subject to slight changes to the mixtures, it remains unclear if the models will perform well, and most notably its ability to learn and generalize.

Researchers have tried different machine learning models and techniques to predict the mechanical properties of cement-based material and even for some, fly ash based geopolymers properties were also modeled.

Ahmad et al. [17] considered and tried a variety of supervised machine learning methods (including decision tree models, complemented by bagging or bootstrapping techniques) to predict the compressive strength of fly ash based geopolymer. Decision tree (DT) models were also considered by Ma et al. [18] to predict the compressive strength of mining waste-based cement. The authors applied other supervised machine learning models, such as support vector machine (SVM) and the random forest (RF), subject to hyperparameters tuning technique and compared the results of all models. The comparison between models is done using well-known model performance metrics such as the correlation coefficient ($R^{2}$) and the root mean square error (RMSE).

Furthermore, Hamie et al. [7] constructed a DT module to predict the pumpability of geopolymers developed for well cementing applications. An additional discrete type model, logistic regression (LR) was applied in order to estimate the probability that a certain geopolymer mixture, subjected to different external properties (time, temperature), will attain a certain desired level of consistency. Other authors, notably Jose et al. [19], have also used similar and alternative discrete type models, such as the probit regression.

With the same spirit of computing the likelihood of an event to happen, Albatayneh et al. [20] have used a mix of parametric and non-parametric models. In fact, classical non-parametric Bayesian regularization artificial neural network (BRANN) and parametric probit regression models were applied to gain insight about the data and the likelihood of each event.

Shaqadan [21] predicted the compressive strength of Ordinary Portland cement using the random forest supervised machine learning model. With the use of bootstrap techniques (with replacement), trees are independently built. The model showed its effectiveness and its ability to forecast compressive strength. The importance of cross validation and hyperparameter tuning methods was important in improving model performance.

Nazari and Pacheco Torgal [22] employed the classical artificial neural network (ANN) models. Using various architecture and hyperparametric tuning (such as backpropagation algorithm, with percentage of absolute error as a stopping parameter, among others), the prediction ability and performance of the models were improved and accurate measures of geopolymer compressive strength results were attained.

Similarly, Nazari and Riahi [23] used adaptive network-based fuzzy inference systems (ANFIS) to predict the water absorption of geopolymers. Based on the root mean square error model performance metric, the authors concluded that the model showed good performance. Moreover, Nazari and Riahi [24] investigated the efficiency of classical neural network, by comparing its results with another model, namely, the gene expression programming (GEP) water absorption predictions. The results showed that the classical ANN outperformed GEP, at the cost of additional tuning complexity, when compared with the latter. Similar work was also conducted by Nazari and Ghafouri Safarnejad [25] who predicted the compressive strength of geopolymer based cement using GEP. Additional exotic machine learning techniques also proved their strong prediction ability. Khursheed et al. [26] predicted the compressive strength of fly ash-based cement using minimax probability machine regression (MPMR), relevance vector machine (RVM), genetic programming (GP), emotional neural network (ENN) and extreme learning machine (ELM). The RVM outperformed the remaining models.

## 3 Experimental data

**Fig. 1:**
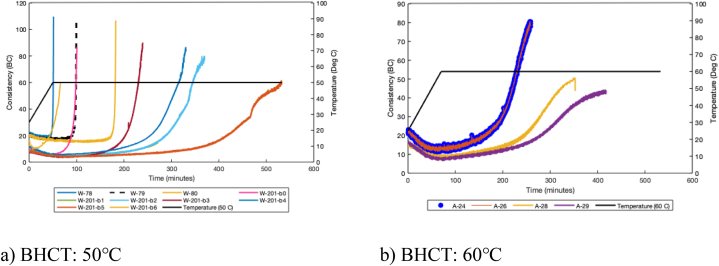
Consistency data of the slurries, for a variety of geopolymer mixtures, at ambient pressure and two different bottomhole circulation temperatures [7,27,31].

Geothermal gradient is a critical parameter in well cementing operations; usually downhole temperatures are divided to bottomhole circulating temperature (BHCT) and bottomhole static temperature (BHST). BHST is the geothermal gradient at a given depth, it is used to study mechanical properties of the hardened material. At a given depth, BHCT is lower than the geothermal gradient due to cooling effect of drilling fluid, it is used for studying pumpability of cement during displacement. In this study, we used 50 and 60 °C as BHCT (See Fig. 1a and b) for intermediate and production casings, respectively. As the slurry is pumped into wellbore, the temperature is ramped up, until it reaches the target temperature (e.g., 50 °C or 60 °C). At the beginning of the experiment, and as both time and temperature values start to increase, consistency decreases until a certain limit. This is regarded as the dissolution phase where reactive aluminosilicates are dissolved and transported. Then, over time, at constant temperature the consistency starts to increase, this is known as oligomerization phase. A third important phase is where the consistency increases at a fast pace. This is visualized in Fig. 1, more specifically in the sudden increase in slope (right angle convexity for some of the slurries) of each mixture, at a certain point in time (different by mixture). This is the polycondensation phase where all oligomers get linked and the slurry solidifies.

When the molar ratio (SiO2/K2O) of the slurry is increased, the polycondensation rate decreases and therefore, the right-angle-set disappears (see test conducted at 60 °C, Fig. 1b), at the expense of a convex shape with an acute angle. This can also be done by use of retarders (e.g., W-201-b5). All the tests have been conducted three times to minimize errors and uncertainties originating from experimental work. The chemistry and mix designs of these slurries have been thoroughly discussed in previous publications [10,[27], [28], [29], [30]].

It is true that all experimental mixtures witness the same chemical phases, however it is also clear from the figure that they do not behave the same way. Under different mixture components, the behavior is not unique, and this will pose challenges in the analytical and modeling part. At the same time, it is an opportunity to test the ability of the models to learn and generalize at the same time.

Table 1 presents the descriptive statistics of the full dataset used in the analysis. The authors aim to focus on the mechanical property of Consistency (as the dependent variable) and all remaining variables as experimental. As it can be seen from the table, the main numerical variables is the consistency, used by engineers to measure the pumpability of the material, and for each mixture design, subject to different temperature gradients, this variable is measured over time and subject to a constant applied ambient pressure. The mix design, however, can be defined by a series of discrete variables that describe the chemical composition of the mixture (rocks, silica, geopolymer hardener, etc. full list is found below), as well as the states of matter (solid/liquid phases). In total, there are fourteen mixtures (Fig. 1) that are used in our analysis. In Table 1, data for all mixtures is grouped and various mathematical models are applied on the grouped data, in other words, one model is built on all mixtures, instead of a parametrizing a set of parameters for each mixture. The reason for that is to increase the dataset and equip the machine learning based models with an opportunity to make causality inference and make predictions based on them. The bigger the number of mixtures used, the better, but of course, this comes at the expense of higher cost, more time spent in laboratories and cutting-edge laboratory machines.

**Table 1:** Descriptive statistics of the data set.

Continuous/numerical variable								
Variable	**Unit**	**Min.**	**Q1**	**Median**	**Mean**	**Q3**	**Max.**	**SD**
Consistency	Bc	2.98	6.99	12.69	17.27	19.63	109.55	15.13
Time	Minutes	0.02	68.42	149.02	166.56	241.91	532.88	166.56
Temperature	Degrees Celsius	25	50	50	51.87	60	60	51.87
Discrete variable								
**Potassium Nitrate**$KNO_{3}$	g	Possible values (0, 1, 2 or 3) and proportions (%) of each (12.3, 34.2, 17, 36.5)						
**Calcium Carbonate**$CaCO_{3}$	g	Possible values (0, 3 or 4) and proportions (%) of each (63.5, 26.5, 10)						
**Aluminum hydroxide**$AlOH_{3}$	g	Proportions (0, 5 or 6) and proportions (%) of each (81, 12, 7)						
K−silicatesolution(pH14)	g	Possible values (77, 79, 82, 85, 88, 90 or 91) and proportions (%) of each (17, 20, 5, 18, 2, 1.5, 36.5)						
K−silicatesolution(pH11.5)	g	Possible values (220, 223, 225 or 228) and proportions (%) of each (1.5, 90.5, 3, 5)						
**Water**$H_{2}O$	g	Possible values (47, 57 or 78) and proportions (%) of each (14, 76.5, 9.5)						
**Zinc Nitrate**$ZnNO_{3}$	g	Possible values (0, 4, 6 or 8) and proportions (%) of each (13, 34, 17, 36)						
Binary variable								
**Sodium Hydroxide**NaOH	g	Possible values (0 or 1) Proportions (%) of each (63.5, 36.5)						
Granite	g	Possible values (350 or 490) Proportions (%) of each (9.5, 90.5)						
Microsilica	g	Possible values (0 or 10) Proportions (%) of each (9.5, 90.5)						
**Ground granulated blast-furnace slag (**BFS**)**	g	Possible values (200 or 350) Proportions (%) of each (90.5, 9.5)						

Note: There are no missing values in the dataset. Total number of observations per variable is 8475.

## 4 Methods/data and model formulation

### 4.1 Classification models

Five binary classification models are used, where the dependent variable takes only two values, either zero or one. The latter designates a consistency value for a certain mixture exceeding a certain threshold, which we will refer to by classification threshold in what follows. These are: Logistic regression, Probit regression, Decision Tree, Random Forest, Support Vector Machine (SVM). Four of these models, predicts the probability/likelihood that the consistency of a certain observation, characterized by a given set of features (Temperature, Time, Mixture composition, etc.), will exceed the classification threshold. The fifth model (SVM) directly predicts the class, instead of computing the probability.

### 4.2 Pre-modeling and data analysis

The analysis starts with a check for multicollinearity. High degree of correlation (≥0.75) between independent variables can cause problems when fitting the model and interpreting the results. A possible solution to this problem would be to remove some of the highly correlated independent variables. Fixing the classification threshold above which the binary dependent variable Y is equal to 1, is an essential step. It is important and we remind our readers that there are three chemical phases that our mixture endure during the experiment (starting with the dissolution phase, oligomerization phase and finally the polycondensation phase). It is noted that the consistency does not increase at rapid pace during the first two phases, see Fig. 1. The polycondensation phase is the third and final phase, where the consistency levels increase to higher levels, until reaching 100 BCE. The higher the level of consistency the thicker the slurry. A slurry that fails to reach that phase, is a slurry that is not in solid phase, thus not a qualified cementitious material.

As mentioned earlier, one can notice that the variety of mixtures used in this study do not share the same behavior. This is simply due to the fact that each mixture has a different mix design (e.g., water content, chemical admixtures, etc.), and also the mixtures are subject to different temperatures. This means that the consistency threshold for each mixture might vary. This threshold can either be estimated graphically, or analytically. The latter analysis, is based on approximating the derivative of function that defines the behavior of the slurry consistency with respect to the temperature, given by a numerical approximation of a second order Taylor expansion. In this context, the derivative of a function f at a point x_i_ is approximated by Equation (1):

(1) $$f^{\prime }\left(x_{i}\right)≅\frac{-f\left(x_{i+2}\right)+4f\left(x_{i+1}\right)-3f\left(x_{i}\right)}{2h}$$

where h is the length of the step between the points xi, x_i+1_ and x_i+2_. The point at which the curve starts to increase excessively, and the concave upward shape starts to take form (concave edge), is the slurry-specific consistency threshold.

Once the thresholds for all mixtures are estimated analytically, the lowest threshold will be selected as the common value for all mixtures at which all observations above it, will be classified as one, and the remaining ones as zero. In our application, the estimated value of the common threshold is 22.38 BCE. Second, a closer look at the dependent variable, consistency, show that on average the proportion of class one observations (consistency value at a certain time and temperature, exceeding the threshold) to the overall number of observations is one fifth. From an empirical probabilistic angle, this would mean, that there is a 20% chance that a random selected observation is classified as one. Finally, and as part of our pre-modeling data treatment, we divide the data into training and testing sets with a ratio of 80/20, respectively. The first set will be used to train the model and the other set for testing purposes.

In an attempt to avoid over fitting, test the ability of the model to generalize and, give accurate predictions for new and untested data, the following steps are applied.

- Applying the classical technique of splitting the data into training and testing set. The model is built on the training set and tested on the other set to assess its ability of generalization. The selection of the latter is based on an iterative cross validation.
- By K folds cross-validation, we are repeating the process introduced in the previous point on K randomly selected training and testing sets, in a sequential way, based on a bootstrapping sampling technique. The predictive power of the model is finally assessed by computing the average of the considered performance metric on all the K testing sets.
- To control the complexity of some models, a regularization parameter was added to the objective (loss) function to penalize complex and deeper models.
- Finally, the main hyper-parameters of the models were tuned in a way to get an optimal result in terms of model performance metric (e.g., R2, pseudo-R2, MSE, confusion matrix, Receiver Operating Characteristic Curve, ROC, plots, Area Under the Curve, AUC, on the testing data). To do this tuning, a grid search technique (limited one, after considering some prior assumptions about the range of possible hyperparameters values, in order not to use all combinations) was applied. The range was subjectively set, based on authors experience. This hyper-parameter tuning step is coupled with the K folds cross-validation technique to ensure that the optimization was done independently from the choice of the underlying data train/test split.

### 4.3 Logistic regression

The first of the two regression models used is the Binary logistic regression. The model is formulated as per Equation (2):

(2) $$P\left(Y=1|\;X\right)=\frac{1}{1+e^{-\left(\beta_{0}+\beta_{1}X_{1}+\ldots +\beta_{p}X_{p}\right)}}$$

where X_i_ are the independent variables, ß_i_ the corresponding parameters and Y, the consistency as the dependent variable. The observation belongs to the positive class (class 1) if its computed probability is above the classification threshold, while it is classified as negative (class 0) if it is below. The mainstream assumptions for parametric regressions are applied in such a model. Assumptions include independence of errors, no multicollinearity between independent variables, and no presence of significant outliers. Independent variables should be carefully selected and can be categorical or numerical.

### 4.4 Probit regression

The second of the two regression models used is the Probit model. It is similar to the model previously described; however, it uses another functional form. The cumulative distribution function of the standard normal distribution is used to model the regression function as per Equations (3), (4):

(3) $$P\left(Y=1|\;X\right)=\Phi \;\left(\beta_{0}+\beta_{1}-X_{1}+\cdots +\beta_{p}\;X_{p}\right)$$

where

(4) Φ(z)=P(Z≤z),Z∼N(0,1)

Similarly, the Probit model returns a score between 0 and 1, which gives the probability of the observation to be classified in the success category.

### 4.5 Classification Decision Tree

Classification Decision Tree is a supervised machine learning algorithm. As its name indicates, decision tree uses a tree diagram of decisions at each node and return the final probability of classification. Decisions and nodes split are created using information gain or entropy metric (measure of impurity). As with the regression models previously described, this model also computes the probability of being classified as success (in our case, it is defined as the probability that the consistency of the mixture exceeds the classification threshold) given certain characteristics (such as pressure, time, temperature, mixture configuration). Multiple hyperparameters are to be considered when growing a tree. In this study the hyperparameters tuned for this model are: complexity, minimum number of observations when splitting a node and a depth parameter that dictates how many splits can happen.

Although decision trees are simple visualization of the variable's relationships and flow, they are easily affected by data noise, large number of variables and large datasets may lead to overfitting issues.

### 4.6 Classification random forest

Another supervised machine learning algorithm is also employed, and it essentially composed of multiple individual independent decision trees. Each one runs independently and without any interaction with the other trees. The predictions of those trees are aggregated to produce the final results. Two hyperparameters are considered when growing the random forest model in our study. Starting with the main parameter that dictates the characteristics of each tree grown and that controls, in a way, how much randomness is added. This parameter controls how many of the explanatory variables shall be considered at each tree whenever a split takes place. The other important variable considered is the number of trees that we intend to grow.

In general, Random Forest are more accurate than Decision Tree algorithm. They are more effective when dealing with missing data and more efficient in resolving the overfitting problem [32].

As mentioned previously, a grid search technique was applied. We consider all possible combinations of the supplied vectors, each vector containing a range (i.e., continuous or discrete) of values that can be attributed for each hyperparameter that needs to be tested against. The model is then computed α number of times, where α represents the total number of possible hyperparameter(s) combinations. The possible range of values for each hyperparameter is subjectively set, based on authors experience gained during the lab testing procedures, as well as the empirical work on data analysis.

Five parameters are tuned for both Decision tree and Random Forest models, and the range of each, is tabulated in Table 2.

**Table 2:** Grid search technique parameters.

*Hyperparameters tuned*	Min value	Max value	Increments	Length
*Decision Tree – complexity*	0.001	0.1	0.01	10
*Decision Tree – number of observations chosen when splitting a node*	60	100	10	5
*Decision Tree – depth parameter*	6	10	1	5
*Random Forest – number of explanatory variables considered at each split*	2	6	1	5
*Random Forest – number of Trees that need to be grown*	50	100	10	6

The model with the best performance on the testing dataset is then selected. The metric used to measure the performance is the coefficient of determination $R^{2}$ for regression-based models and the accuracy extracted from a confusion matrix for classification-based models.

The hyper-parameter tuning step is coupled with the K folds cross-validation technique to ensure to lower the risk of possible overfitting. K is set to ten, therefore our dataset is split into 10 equal subsets, trained on 9 of them, randomly selected, and the remaining subset will be used to evaluate the model performance. These steps are repeated three times, and finally, the mean of all prediction errors is assumed to be the model performance score (while relying on the coefficient of determination and accuracy measures as performance metrics).

### 4.7 Support vector machine (SVM)

The final supervised algorithm used is the SVM. Unlike the previous models, instead of computing a probability, the class to which the observation belongs is directly predicted. SVM are classifiers that rely on two key ideas, the first being, the ability to deal with nonlinear discrimination problems, and the second key feature is to reformulate the classification problem as a quadratic optimization problem. To be able to deal with cases where the data is not linearly separable, the idea of SVMs is to transform the space of representation of the input data into a space of higher dimension, in which it is likely that there is a linear separation. Mathematically, this is achieved thanks to the kernel function, which must respect some mathematical conditions, and which has the advantage of not requiring the explicit knowledge of the transformation to be applied for the change of space. The selection of the appropriate Kernel function can be considered as the main hyperparameter to be optimized in the SVM.

### 4.8 Multiclass/multi-threshold classification random forest

In this section, a general type of classification is considered, the multiclass classification. Instead of having one common threshold value for all mixtures, and to add flexibility to the model, multiple classes are considered. From a modeling perspective, the dependent variable Y $\in \left\{\alpha_{1},\alpha_{2},\alpha_{3},\alpha_{4},\alpha_{5}\right\}$ has five possible thresholds/classes, each indicating a value/class of consistency.

$$\alpha_{1}\leq 22.38\;Bc$$

$${22.38<\alpha }_{2}\leq 27.38\;Bc$$

$${27.38<\alpha }_{3}\leq 32.38\;Bc$$

$${32.38<\alpha }_{4}<37.38\;Bc$$

$$\alpha_{5}\geq 37.38\;Bc$$

Several algorithms could be used for a multiclass classification, such as k-Nearest Neighbors, k-SVM, decision trees, random forest, etc. In this study the random forest classifier is used. Its use is justified by the good prediction performance of this model for binary classifier models explained above (as will be shown in the results section). Similar to data treatment done in the binary classifier, we will decompose our dataset into training and testing sets with a ratio of 80/20, respectively. The model is built on the training set and tested on the testing set. The confusion matrix will be used to assess the performance of the model.

### 4.9 Regression models

Now, in order to go beyond just classifying each observation and force it to belong to two or multiple classes of consistency, the next step is to predict the exact value of the consistency for a given observed values of the independent variables. To do that we will be applying five different regression-based models where the dependent variable is the value of the consistency while relying on the same choice of independent variables as the one used as for the classification models. These are: Multi-Linear Regression, Decision Tree, Random Forest, Support Vector Regression (SVR) and the Artificial Neural Network (ANN). All these models will be predicting the exact value of the consistency of a certain observation, characterized by a given set of features (Temperature, Time, Mixture composition, etc.).

Here also we will be adapting the same techniques to avoid overfitting, optimize hyperparameters of each model and select the model with the highest performance on testing data. As a quick reminder here, the list of these fine-tuning techniques: divide data into training and testing set; apply a K-folds cross validation based on a bootstrapping sampling technique; optimize the hyperparameters of the different models by maximizing a performance metric computed on testing data.

#### 4.9.1 Multilinear regression

The multilinear regression model is one of the most popular statistical learning supervised models used for estimation and prediction purposes. The model is formulated as seen in Equation (5):

(5) $$Y=\beta_{0}+\beta_{1}X_{1}+\ldots +\beta_{p}X_{p}+\varepsilon$$

where X_i_ are the independent variables and β_i_ the corresponding coefficients. ε the residual part of the model containing the information on the variation of the dependent variable Y that cannot be explained by the dependent variables. Here also, the mainstream assumptions for parametric regressions are applied in such a model. Note that only statistically significant independent variables are selected based on a student statistical test.

#### 4.9.2 Decision tree and random forest for regression

The same mathematical formalization of both models as in the classification context is applied for continuous variables, the only difference is that final nodes (decision nodes) of a tree are respectively affected by the mean of the observations that belong to these nodes instead of computing the probability of a given class. Same hyperparameters are to be optimized as in classification.

#### 4.9.3 Support Vector Regression

The Support Vector Regression (SVR) is based on the same theoretical ideas as the SVM with the difference that the output is a real variable. The principle is the same (i.e., optimization of the margins of a selected hyperplane), but with a parameter of tolerance added to the margins in order to take into consideration that we are in the context of regression and not classification. Training SVR is based on numerical optimization techniques with computational level of complexity higher than the classification SVM case.

#### 4.9.4 Artificial neural network (ANN)

Deep learning models showed a good accuracy over recent years when attempting to forecast continuous dependent variables in many different fields of application. In this paper, an ANN is designed to forecast the consistency level for different combinations of the underlying dependent variables. The architecture of the ANN (number of layers, number of nodes within each layer, activation function, loss function, etc.) is optimized in a way to get maximum accuracy on the testing dataset after a cross validation process. All the weights of the ANN are estimated based on the classical backpropagation gradient decent technique. The post-hyperparameter tuning for the ANN model is also based on a grid search technique, whereby the following hyperparameters have been used: activation function (ReLU), number of neurons (13 in first layer and 11 in the second one), number of hidden layers (2), batch and epochs sizes (50 and 200), optimizer (Adam).

## 5 Result & discussion

### 5.1 Classification models

**Fig. 2:**
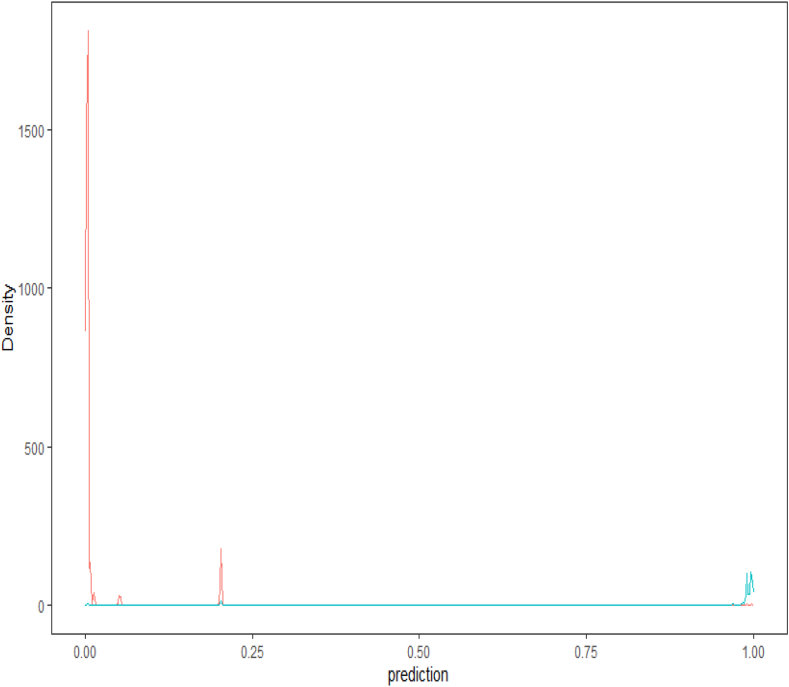
Probability density function of the Decision Tree Model.

**Fig. 3:**
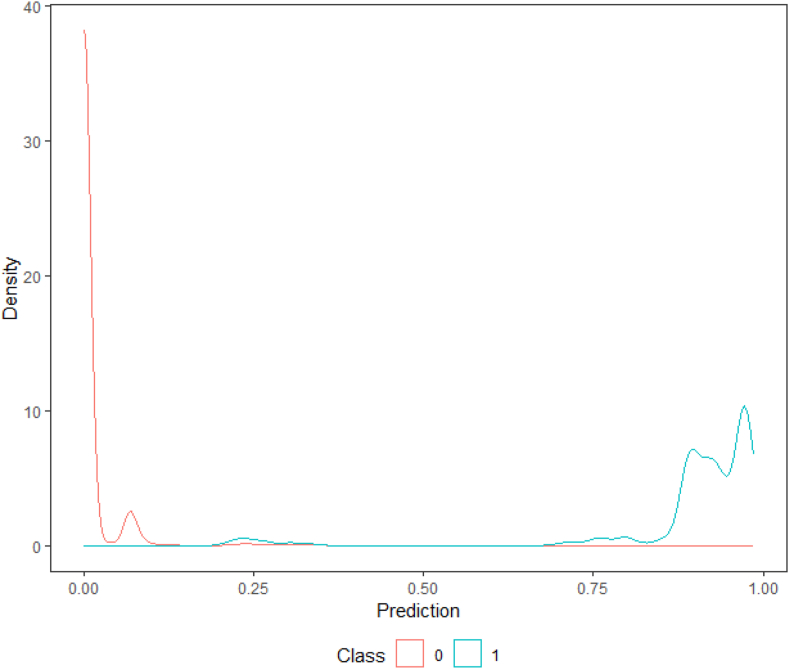
Probability density function of the Random Forest Model.

A first evaluation of the model's performance is done visually by plotting the density function of the predicted probabilities on the testing data set. In an ideal performing model's results scenario, we would expect the two density functions (class zero and class one) to be skewed apart: the negative density plot (class zero) skewed to the left, and the positive one (class one) to the right. Although there is a low ratio of observations that are classified as ones, yet all classification models are producing accurate predictions (see Table 3), more specifically both hierarchical-based models are producing the most accurate results (decision tree and random forest, see Fig. 2, Fig. 3).

**Table 3:** Results for all classification models.

	Logistic Regression	Probit Regression	Decision Tree	Random Forest	SVM
Area Under the Curve, AUC	0.963	0.964	0.991	0.994	NA
K- Cutoff	0.460	0.420	0.87	0.34	NA
Total cost	94,450	96,200	22,200	13,900	NA
True positive	6455	6390	6712	6624	6696
False positive	277	342	20	38	169
False negative	334	310	101	84	42
True negative	1409	1433	1642	1729	1574
Sensitivity	0.960	0.950	1	0.98	0.975
1- Specificity	0.190	0.180	0.060	0.01	0.026
Accuracy	0.928	0.923	0.986	0.985	0.975

**Fig. 4:**
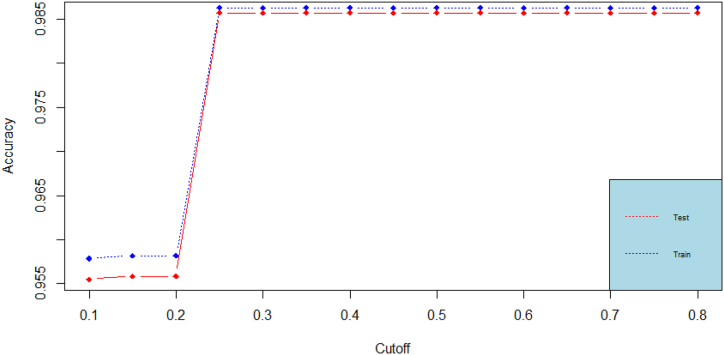
Accuracy plots for the decision tree classification model.

**Fig. 5:**
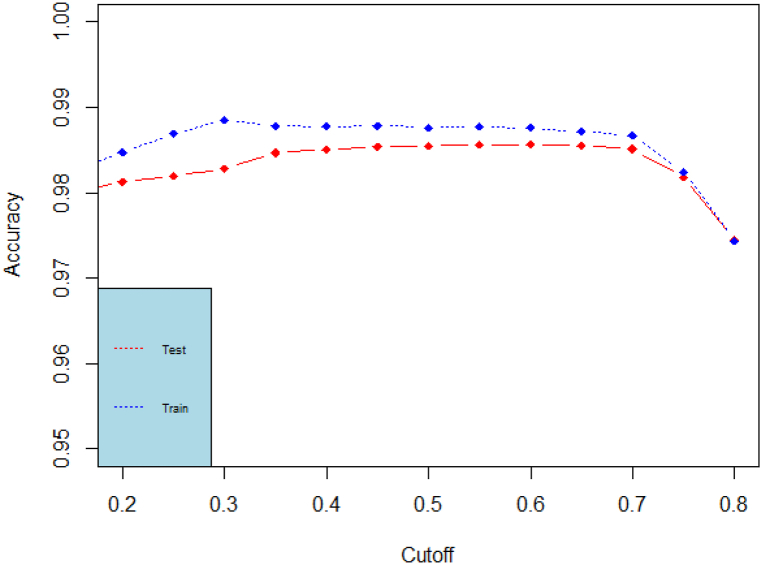
Accuracy plots for the random forest classification model.

We then move, to computing the accuracy, which is the proportion of correct predictions over total predictions (for both classes zero and one). The accuracy ratio is computed for various probability cutoffs. The accuracy for all models keeps on increasing rapidly (especially in the probability cutoff range c∈[0.1,0.6]). It goes down afterwards in the random forest model. The accuracy plots for the best performing models (decision tree and random forest) are found in Fig. 4, Fig. 5. Accuracy by its own is not a sufficient metric for model performance comparison.

Correct predictions are divided into two subgroups (True negative and true positive) and each model shall perform well in both. Increasing the number of true negative (TN ∼ model correctly predicts the observations originally classified as zero), at the expense of a lower number of true positive (TP ∼ model correctly predicts the observations originally classified as one) is not always efficient. In other words, the number of FP (False Positive) decreases at the expense of FN (False Negative).^1^ Of course, the probability cutoff selection also affects these numbers. As mentioned in the previous section, on average the proportion of class one observations to the overall number of observations is one fifth. Acknowledging the fact that most of the observations are classified as zero and that our model is biased towards predicting more zeros than ones, a cost function will be introduced, and it is considered that it is twice costlier to fail in predicting a TP (also called False Positive ∼ FP), than to fail in predicting an TN (otherwise known as False Negative ∼ FN).

For that, the ROC curve measurement is used. The ROC curve plots the sensitivity rate (otherwise known as the True positive rate, TPR, and is defined as follows, $\frac{TP}{TP+FN})$ vs 1-specificity rate (otherwise known as the False Positive Rate, FPR, and is defined as follows, $\frac{FP}{TP+FN})$. A highly sensitive rate means that there are few FN results. A highly specificity rate means there are few FP cases (high TN). Since our model is biased towards predicting class of zero, we prefer high level of sensitivity and an acceptable level of specificity. The ROC curve will help visualize this trade-off: the point closer to the northwest corner corresponds to an optimal cutoff that maximizes sensitivity and specificity. Not only that, but the cutoff should also minimize the total cost already defined for the FP/FN tradeoff.

**Fig. 6:**
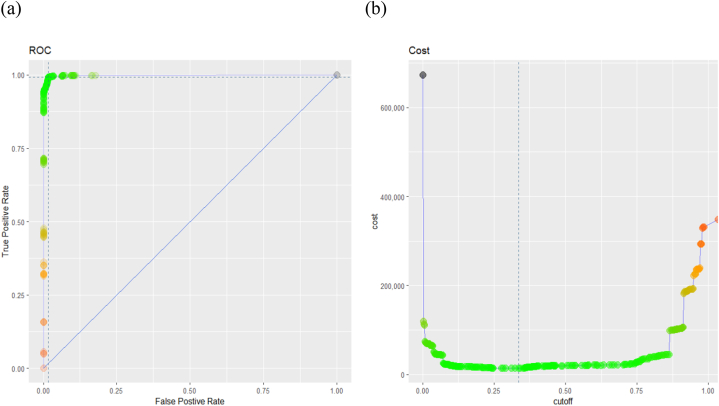
ROC and cost function plots for the random forest model on the testing data. (a) The performance of the Random Forest classification model at all classification thresholds; (b) the performance of the model over the full range of thresholds, while also accounting for the misclassification costs.

**Fig. 7:**
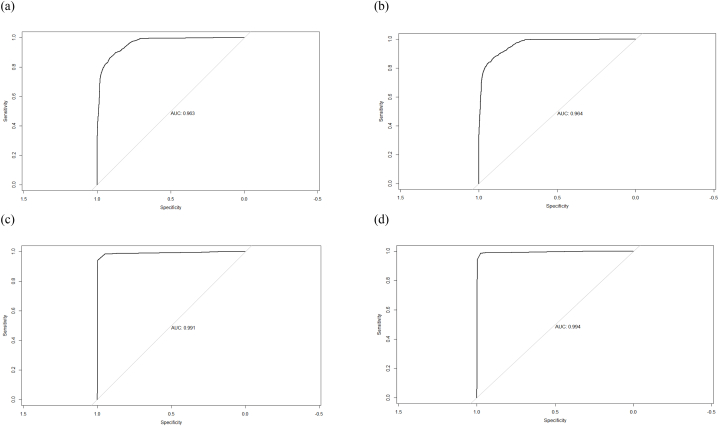
ROC curves for all classification models. (a) Logistic regression; (b) probit regression; (c) decision tree; (d) random forest.

In Fig. 6, the plot on the left represents the ROC curve, while the one to the right is the total cost function. The green zone in the area of the cost function corresponds to a low-cost range; the same color is labeled on the ROC curve as well, whereby, the number of false positives and negatives is almost zero, thus producing values with high TPR and low FPR, an indication of good performing models. The ROC curves for all models are shown in Fig. 7 below.

**Fig. 8:**
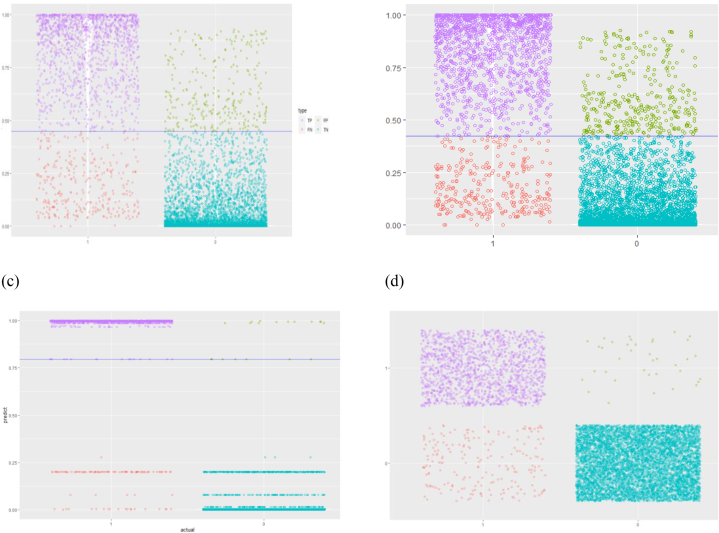
Confusion for models (Logistic Regression, Probit, Decision Tree, SVM). (a) Logistic regression; (b) probit regression; (c) decision tree; (d) random forest.

**Fig. 9:**
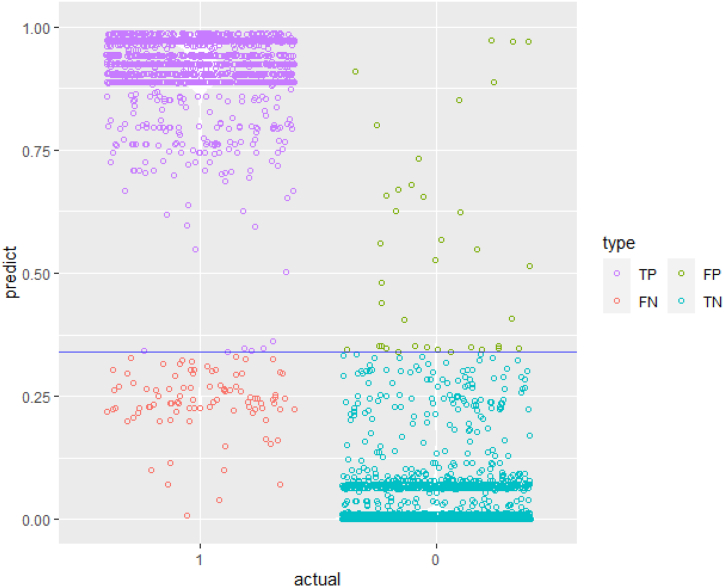
Confusion matrix for the random forest model (One threshold).

The confusion matrices for all models, each computed with the optimized probability cutoff, are shown respectively in Fig. 8, Fig. 9.

More Results are found in Table 3.

It can be deduced from the results shown in Table 3, that the Decision tree and random forest models have the highest AUC, sensitivities, accuracy, and at the same time lowest FPR. As decision tree and random forest have almost the same performance over all the considered metrics, we added an additional criterion to discriminate between both models, which is the computational complexity, normally higher in the random forest. Hence, based on all the above, decision tree model will be selected for the classification part of the application.

False positive refers to the observation(s) where the model(s) fail to predict as negative (originally classified as 0), while false negative refers to the observation(s) where the model(s) fail to predict as positive (originally classified as 1). Therefore, if the model results indicate unjustifiably that the consistency target has been reached (false positive), then, there is a critical risk incomplete placement of the geopolymer slurry, or the slurry will not be able to develop strength. The other scenario, whereby the model incorrectly indicates that the consistency has not been reached should also be given serious considerations. Although the signal/outcome points in a wrong direction, however, the adverse consequences of false negative are proportionally much lower when compared with the other scenario.

While it is true that statistical predictive model risk is defined by wrong and inaccurate predictions (both false negative and false positive), however, the inherent risk of a well cementing activity goes one dimension further and shall be defined in failing to reach the polycondensation phase (false positive). In such a situation, the consequences of poor detection will eventually lead to a huge risk, endanger the plug integrity, and thus lead to short-term repercussions, as well as possibly classifying the whole well integrity operation as compromised.

**Fig. 10:**
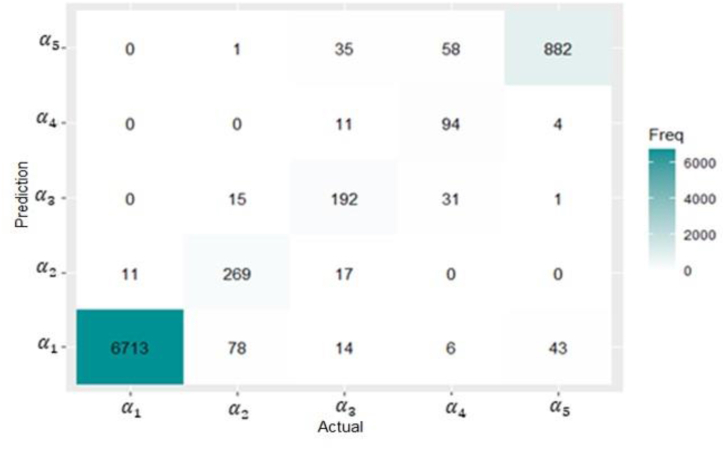
Confusion matrix for the best performing model (multi-thresholds).

Finally, we will apply the multiclass classification using the random forest model and investigate the performance of the model on our data. As for the binary classifier, the dataset is decomposed into training and testing sets with a ratio of 80/20, respectively. The model is built on the training set and tested on the testing set. The confusion matrix result is shown in Fig. 10.

The diagonal entries of this matrix show the frequency of the correctly identified predictions for each class. The non-diagonal entries represent the frequency of incorrectly identified prediction for each class. For instance, the total number of testing observations originally classified as $\alpha_{2}$ class is 363, and the model has accurately classified 269 of them, and failed to classify 94. According to our model, 78 of the wrongly classified observations, are found in class $\alpha_{1}$. The global accuracy of the model is 0.96, it implies good model performance.

### 5.2 Regression models and continuous models

Based on the K-folds cross validation analysis coupled with hyper-parameters grid-search optimization techniques of the different models, we present the results generated by the best hyper-parameters selection of each model. The average $R^{2}$ per model are represented in Table 4.

**Table 4:** Average $R^{2}$ for the models tested in this study.

Models	Average $R^{2}$
Multi-linear Regression (MLR)	0.53
Decision Tree	0.95
Random Forest	0.78
SVR	0.79
ANN	0.92

Hence, the model that produce the optimal results is the Decision Tree. In fact, linear based models (MLR, and SVR) are to be rejected because the relationship that we aim to predict, and model cannot be explained with models that heavily rely on linear model assumptions and that possibly explains why the non-parametric and non-linear machine learning models perform better. The p-values of student statistical test applied for the MLR model showed that all the independent variables are statistically significant and are providing information regarding the variability of the consistency, yet despite this, the model is not able to learn and generalize.

**Fig. 11:**
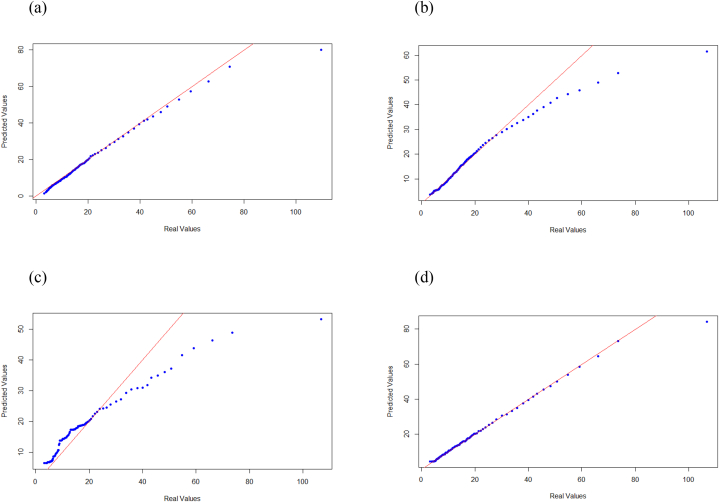
QQ plots (predicted vs. observed) for all continuous models. (a) Artificial neural network; (b) support vector machines; (c) random forest; (d) decision tree.

With the aim to ameliorate the results of the MLR, a first solution is to project our observations in a higher dimensional space, as in the SVR case. It is clear that this technique is increasing significantly the quality of prediction, however it is still not the best scenario to consider. On the other hand, the fact that the random forest is not considering all possible combination of variables to build each of the forest trees, is impacting the performance of the model and its ability to generalize on the testing set. In fact, the subset of independent variables chosen for each of the forest trees are selected randomly, then decisions and nodes split are created using information gain or entropy metric. A comparative parity plots are visualized in Fig. 11, to illustrate the performance of each statistical model. The purpose of these plots is to visualize the predicted vs. real data, by mapping the two sets of quantiles against one another. When both point coordinates have similar distributions, then these points shall form a straight line (red line shown in the above figure). This is obviously the case for the Decision Tree model.

Finally, if the random forest algorithm often chooses some variables, and splits are taken based on those variables, because of the large drop in node impurity, it stands to reason that those variables are important. In fact, the variables that are used more often in the Radom Forest, constitute a subset of all independent variables, knowing that in the MLR, all independent variables were statistically significant.

This is not the case for the decision tree model, which is considering all the independent variables (similar to the MLR) to get the optimal tree and, at the same time, has the capability of explaining nonlinear relationship has to be considered (this is shown in the high R2 metric, compared with other models). The good performance of the ANN comes at the expense of high computational complexity. Finally, one can conclude that the decision tree is preferred because of its high model performance empirical results, compared with other models, and because unlike other machine learning models (including ANN), it provides a graphical, simple and intuitive way to understand what the algorithm does.

## 6 Conclusion

This study has shown that the use of mathematical and statistical models can provide an effective and efficient way of predicting the behavior of geopolymers under various conditions. From the results, it was found that the decision tree and random forest models had almost the same performance level over all considered metrics, but the decision tree model was chosen based on its lower computational complexity.

In general, one can say that non-parametric and non-linear machine learning models perform better in explaining the hidden behaviors of geopolymers dataset especially so, when compared with MLR-based models. To improve the results of the MLR, a first solution that is envisaged is to project observations in a higher dimensional space as suggested by SVM/SVR models. Although this technique significantly improves the quality of prediction, other models (decision tree and ANN) were shown to be able to ameliorate much more the performance.

The decision tree model considers all independent variables and has the capability of explaining non-linear relationships. This is validated with the high R2 metric of the model, when compared with other models. The decision tree model has high model performance empirical results, has lower computations complexity and at the same time has an intuitive/simple way to understand the algorithm. On the other hand, the random forest does not consider all possible combinations of variables to build each of the forest trees, impacting the model's ability to generalize on the testing set.

In conclusion, the decision tree model is the optimal model for modeling the pumpability of geopolymers, as it provides high model performance, simplicity, and interpretability. Non-parametric and non-linear machine learning models can outperform linear models in this context.

## Author contribution statement

Anis Hoayek, Hassan Hamie: Analyzed and interpreted the data; Contributed reagents, materials, analysis tools or data; Wrote the paper.

Mahmoud Khalifeh: Conceived and designed the experiments; Performed the experiments; Wrote the paper.

Bassam El-Ghoul, Rania Zogheib: Contributed reagents, materials, analysis tools or data.

## Data availability statement

Data included in article/supplementary material/referenced in article.

## Declaration of competing interest

The authors declare that they have no known competing financial interests or personal relationships that could have appeared to influence the work reported in this paper.

## Figures and Tables

**Figure:**
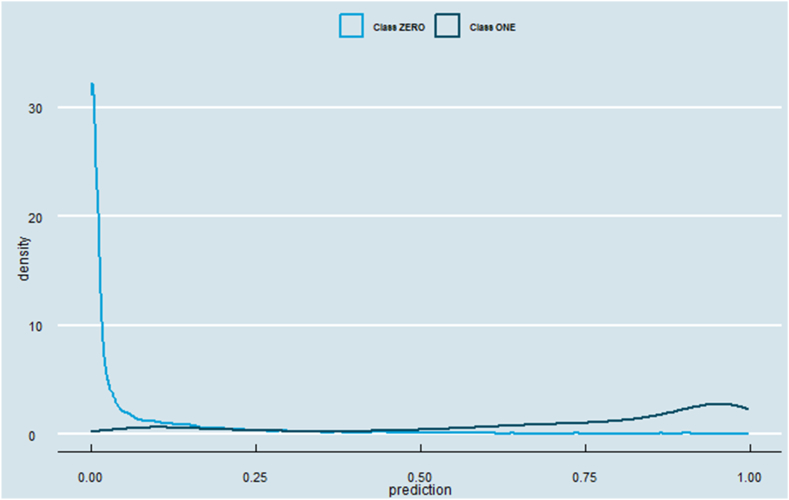


**Figure:**
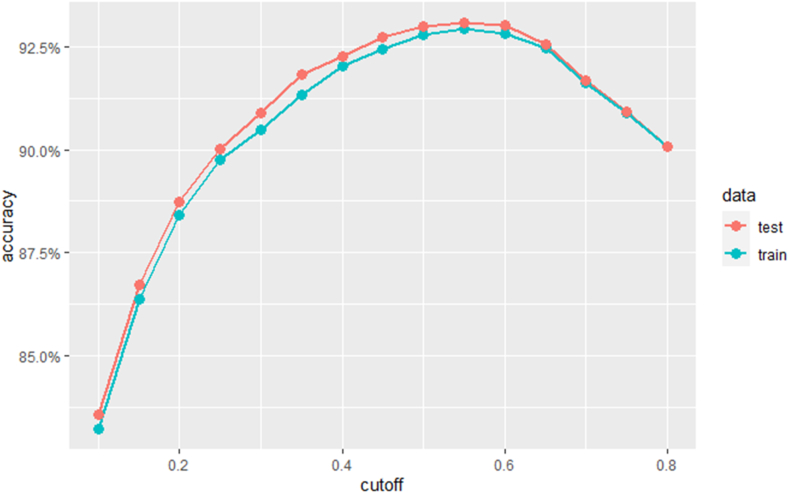


**Figure:**
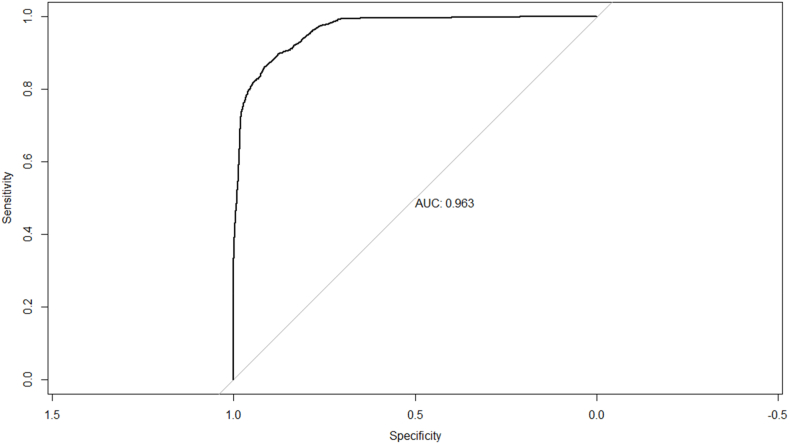


**Figure:**
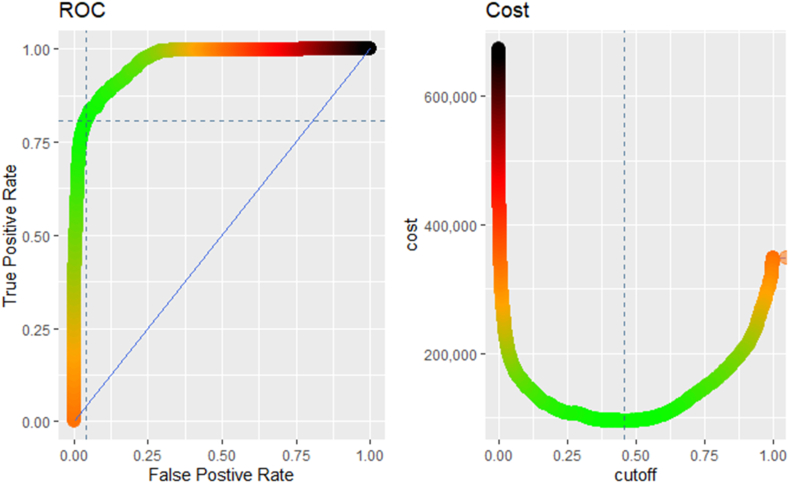


**Figure:**
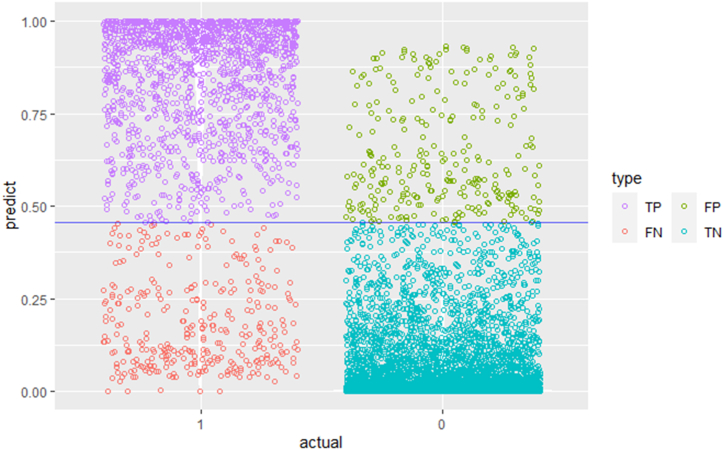


**Figure:**
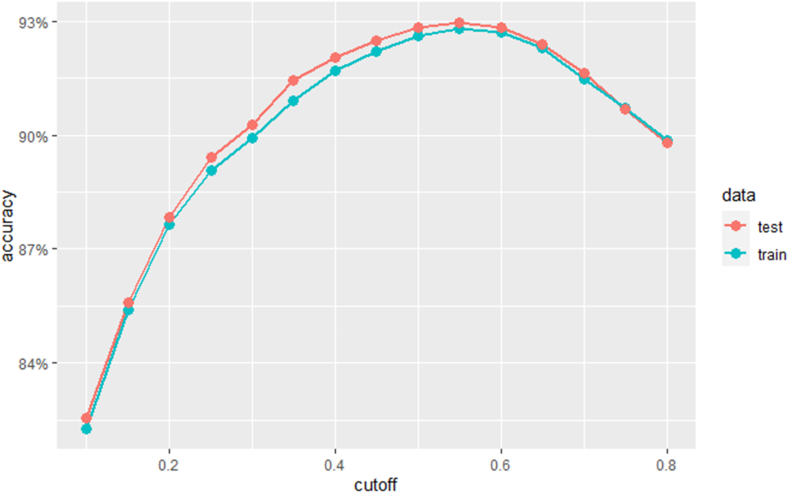


**Figure:**
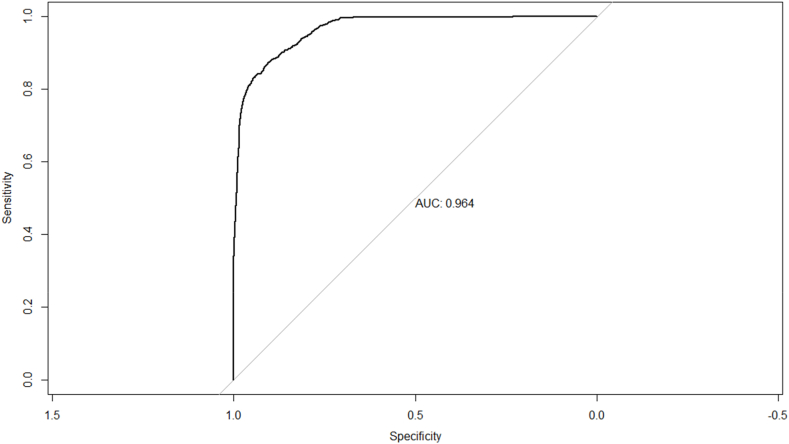


**Figure:**
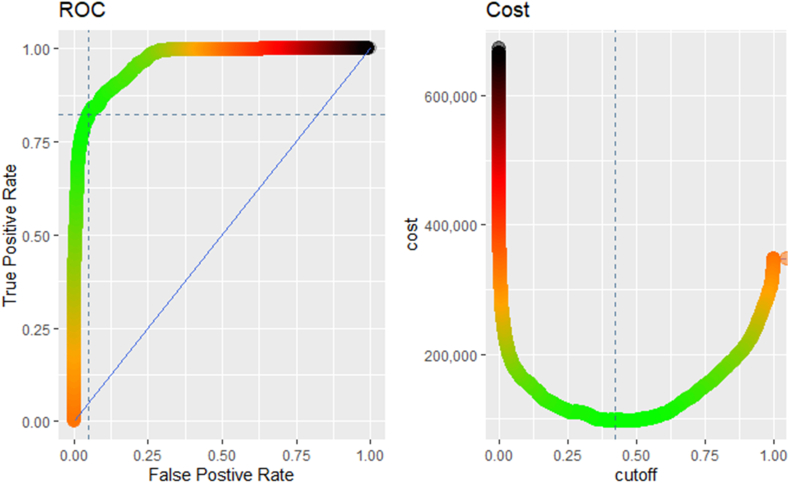


**Figure:**
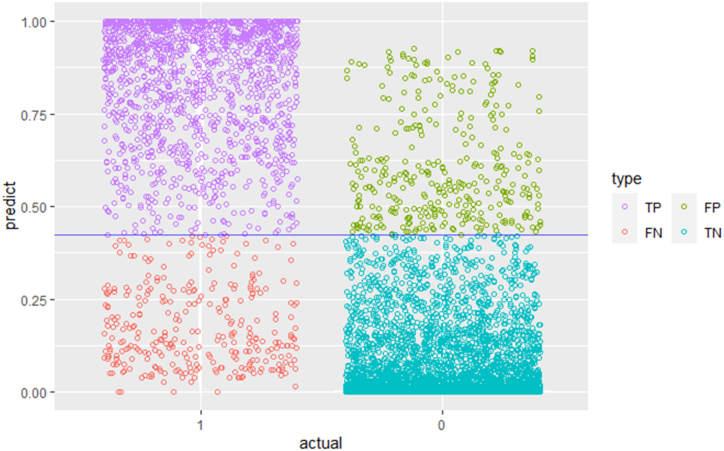


**Figure:**
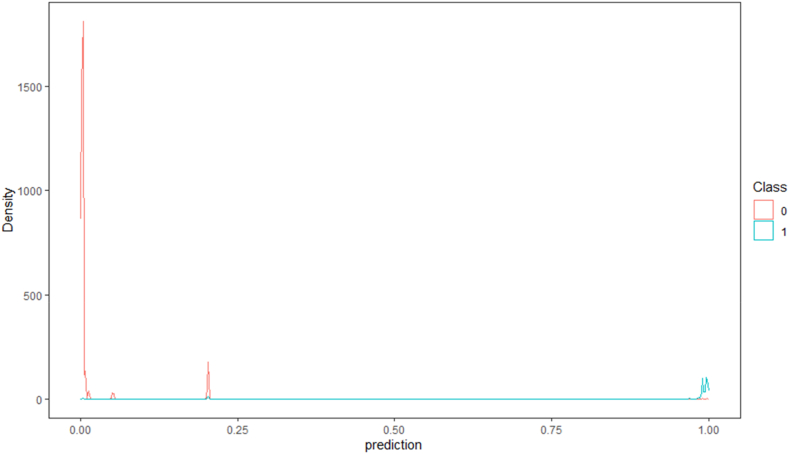


**Figure:**
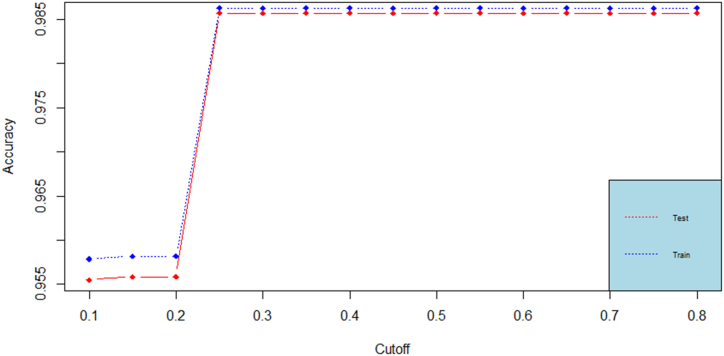


**Figure:**
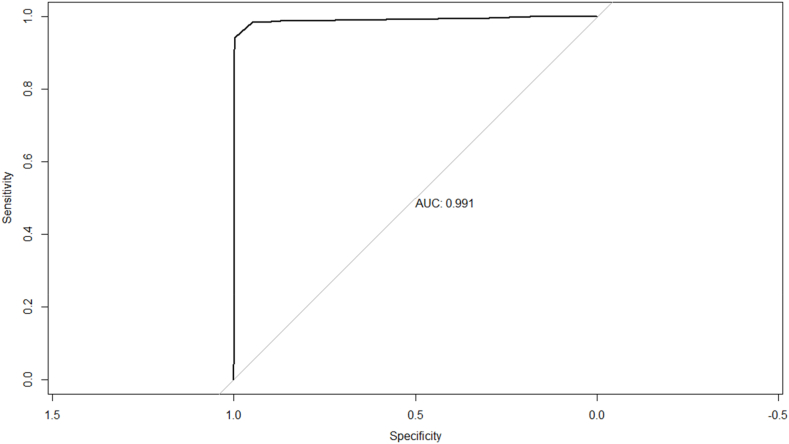


**Figure:**
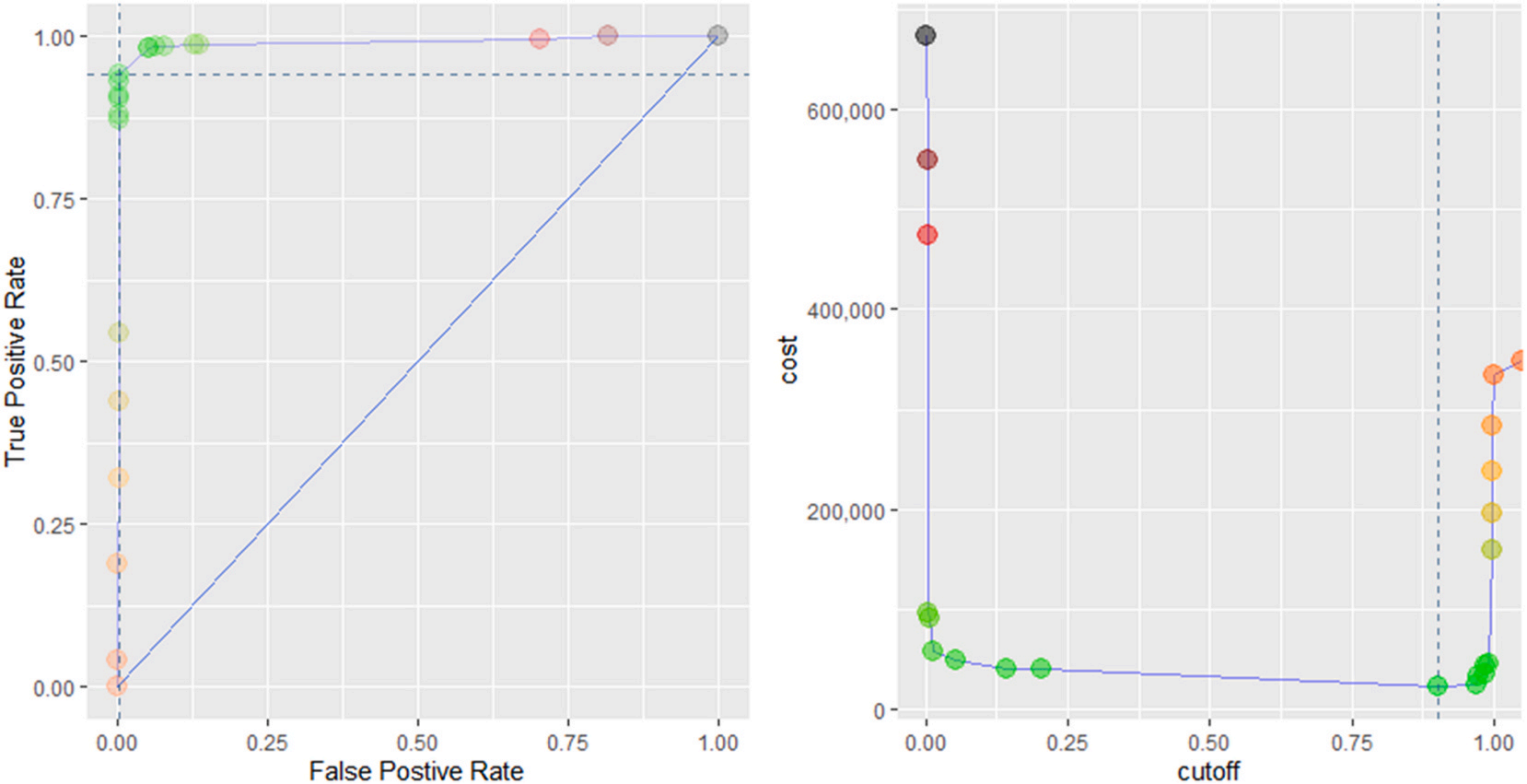


**Figure:**
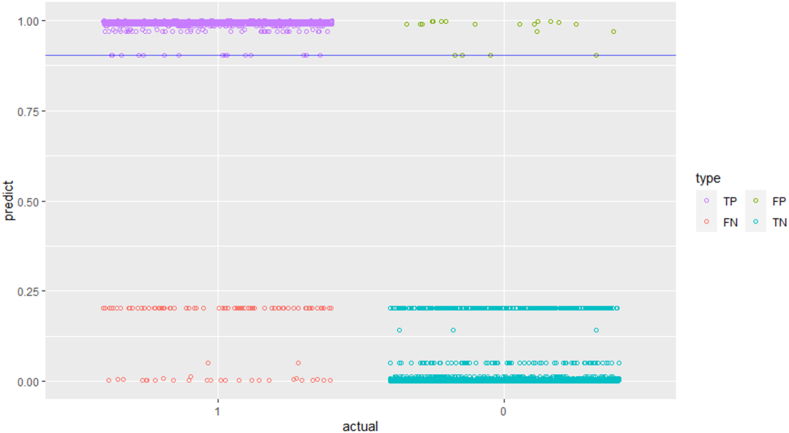


**Figure:**
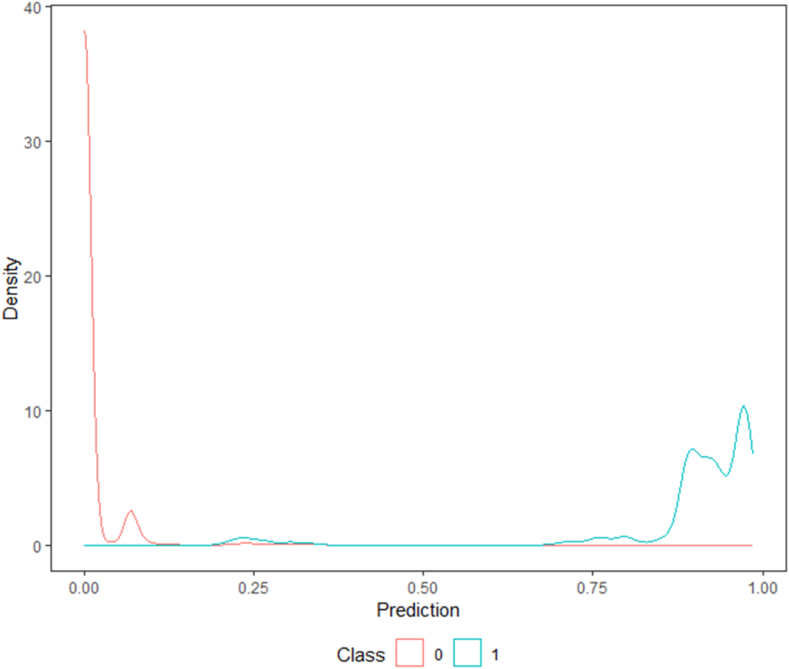


**Figure:**
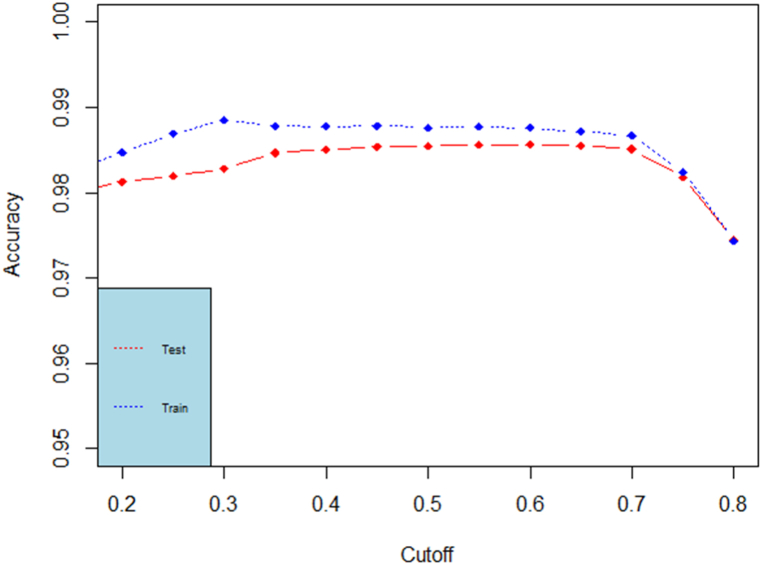


**Figure:**
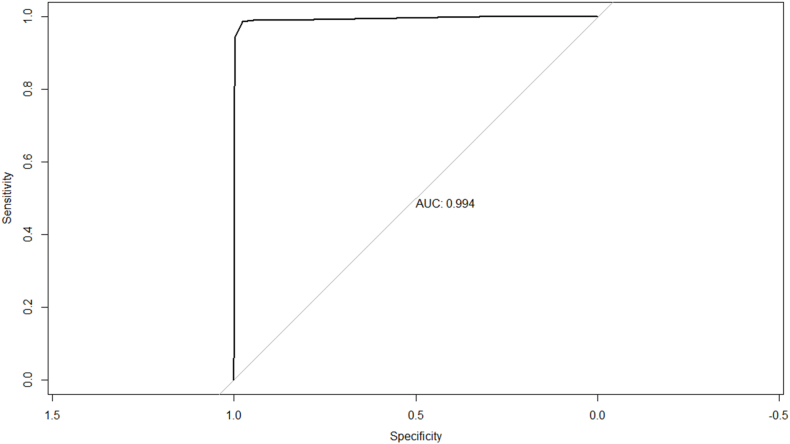


**Figure:**
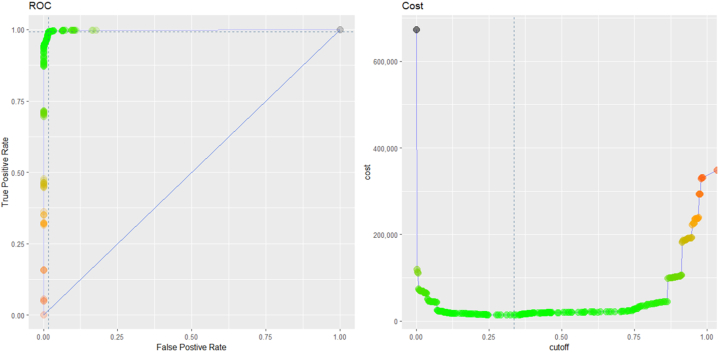


**Figure:**
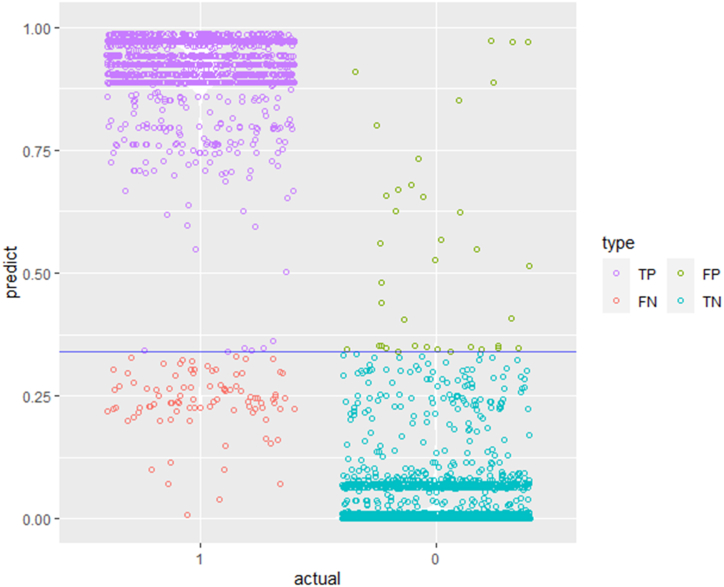


**Figure:**
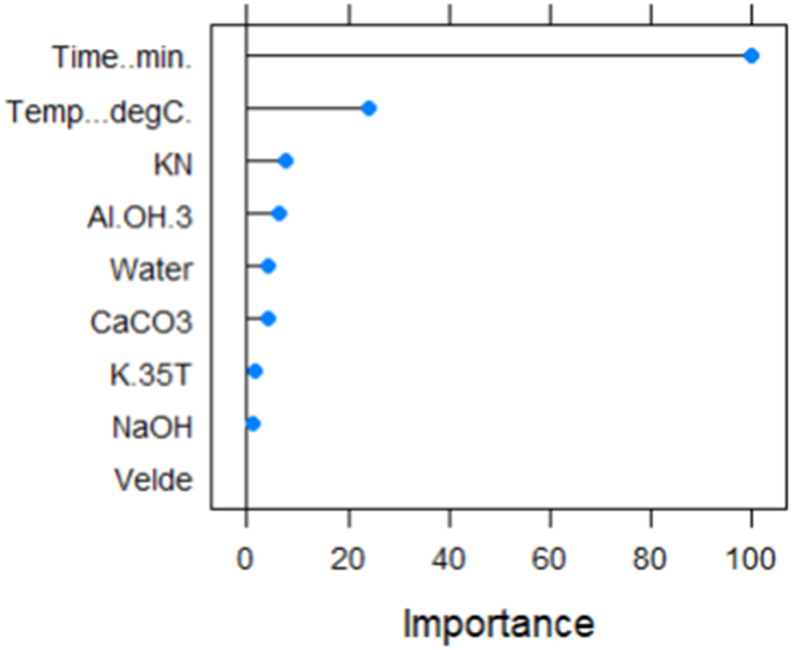


**Figure:**
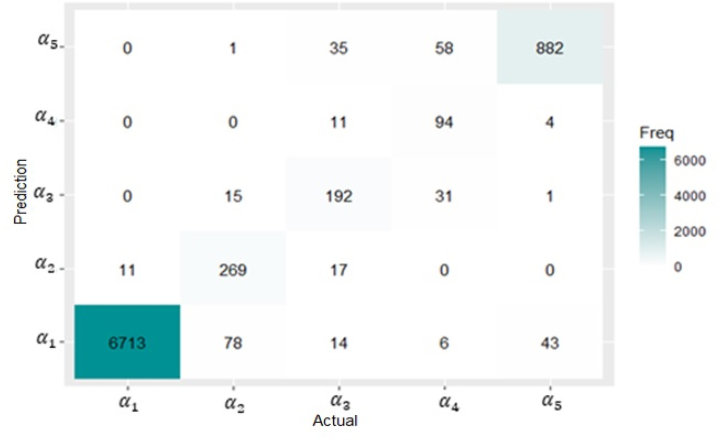


**Figure:**
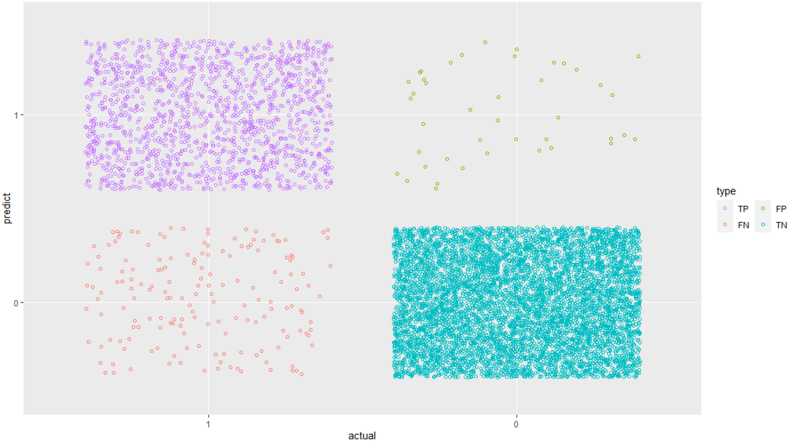


## Appendix

### Logistic Regression plots

Probability density function.

Accuracy plots.

ROC Curve.

Confusion Matrix.

### Probit regression plots

Accuracy plots.

ROC Curve.

Confusion Matrix.

### Decision tree plots

Probability density function.

Accuracy plots.

ROC Curve.

Confusion Matrix.

### Random forest plots

Probability density function.

Accuracy plots.

ROC Curve.

Confusion Matrix.

Random Forest feature importance.

Multiclass classification.

### SVM plots

Confusion Matrix.

## Footnotes

1 False positive/Negative are the observations that the models fail to predict as negative/positive.
